# P20 Identification and detailed bio-evaluation of malabaricone B as a potent antistaphylococcal lead

**DOI:** 10.1093/jacamr/dlac053.020

**Published:** 2022-05-31

**Authors:** Grace Kaul, Neethu Sivadas, K V Radhakrishnan, Sidharth Chopra

**Affiliations:** 1 Division of Microbiology, CSIR-Central Drug Research Institute (CSIR-CDRI), Lucknow 226031, Uttar Pradesh, India; 2 Academy of Scientific and Innovative Research (AcSIR), Ghaziabad- 201002, India; 3 Chemical Sciences and Technology Division, CSIR-National Institute for Interdisciplinary Science and Technology (CSIR-NIIST), Thiruvananthapuram 695019 Kerala, India

## Abstract

**Background:**

The dearth of antimicrobials to treat MDR infections has led to search for novel moieties with potent activity. The most suitable compounds have been derived from mainly natural sources. In this context, the current study focused on identification and determination of antistaphylococcal activity of malabaricone B (MB), one of the naturally abundant secondary metabolites (malabaricones A–D) present in the Myristicaceae family. Malabaricones have previously been reported to possess various biological activities including antimicrobial activity but prospects of its effective utilization against *Staphylococcus aureus* infections in particular have not been explored in detail to the best of our knowledge.

**Methods:**

Malabaricones (A–D) were screened against an ESKAPE pathogen panel via broth microdilution assays. The hit compounds were then tested against Vero cells via MTT assays to determine their selectivity index. As the next step, their activity against clinical drug-resistant strains was evaluated. Further, time–kill kinetics against *S. aureus* were determined along with the ability to synergize with approved drugs via chequerboard assays, which was further validated via combination time–kill kinetics assays. The hit compound's *in vitro* post-antibiotic effect (PAE) and propensity to generate resistance to *S. aureus* was determined. Its activity against intracellular *S. aureus* in a J774 macrophage model of infection and ability to eradicate pre-formed *S. aureus* biofilms *in vitro* was ascertained. Finally, the hit compound's *in vivo* efficacy was determined in a murine neutropenic *S. aureus* thigh infection model.

**Results:**

Amongst the hits identified, MB demonstrated a potent *in vitro* antistaphylococcal profile including equipotent activity against clinical MDR *S. aureus* and *Enterococcus* sp. with MIC 1–2 mg/L. MB exhibited concentration-dependent bactericidal activity with complete killing at 5 × MIC within 15 min of exposure with no regrowth up to 24 h. MB also displayed PAE of ∼22 h at 10 × MIC. It also has very low propensity for generation of resistance to *S. aureus* ATCC 29213. MB significantly eradicated pre-formed *S. aureus* biofilms and reduced intracellular *S. aureus* in the J774 macrophage infection model as well. MB strongly synergized with gentamicin at 1 × MIC *in vitro* with complete killing at 24 h as compared with untreated *S. aureus.* MB also possessed significant *in vivo* activity against *S. aureus* at 50 mg/kg BID dose with ∼0.5 log_10_ cfu/g reduction in thigh compared with untreated mice.

**Conclusions:**

MB demonstrates the potential to be further developed for infections caused by *S. aureus* owing to its excellent *in vitro* and *in vivo* activity.

